# Silica Aerogel in Microfluidic Channels: Synthesis, Chip Integration, Mechanical Reinforcement, and Characterization

**DOI:** 10.1021/acsomega.4c05019

**Published:** 2024-09-23

**Authors:** Ana Luiza Silveira Fiates, Renato S. M. Almeida, Michaela Wilhelm, Kurosch Rezwan, Michael J. Vellekoop

**Affiliations:** † Institute for Microsensors, -Actuators and -Systems (IMSAS), University of Bremen, Bremen 28359, Germany; ‡ Microsystems Center Bremen (MCB), 28359Bremen, Germany; § Advanced Ceramics, University of Bremen, 28359Bremen, Germany; ∥ MAPEX Center for Materials and Process, University of Bremen, 28359Bremen, Germany

## Abstract

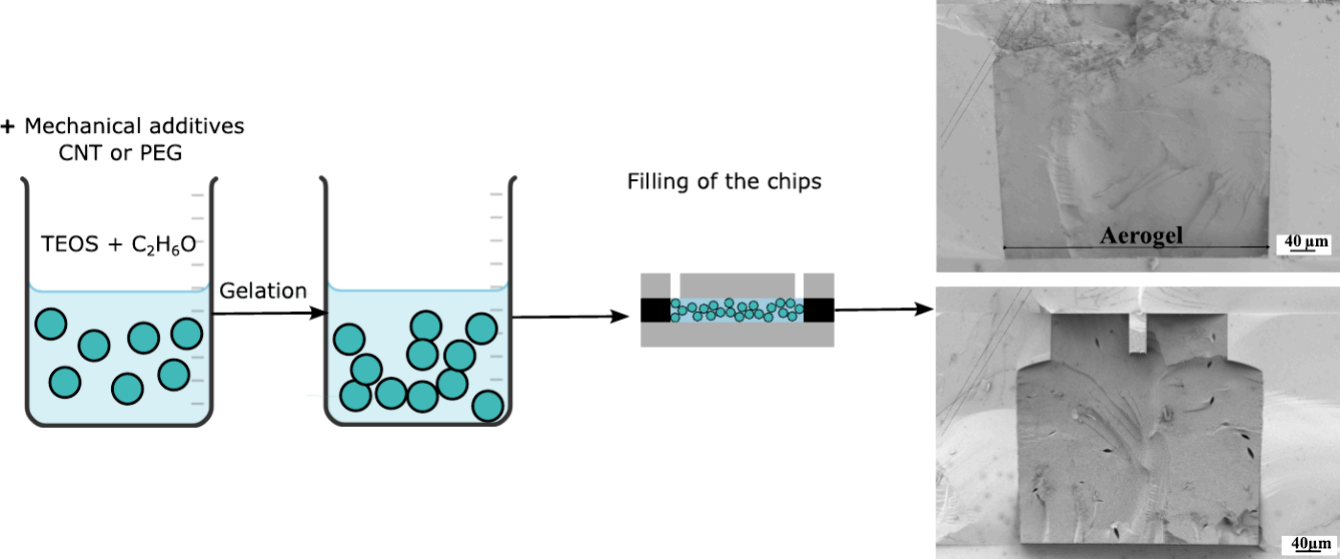

Silica aerogels are highly porous materials with unique properties such as high specific surface area, high thermal insulation, and high open porosity. These characteristics make them attractive for several applications in closed microfluidic channels such as BioMEMS, catalysis, and thermal insulation. However, aerogel-filled microchannels have not been reported in the literature yet because of the complexity of creating a process that controls the integration, shrinkage, and mechanical stability of these materials inside a closed channel. In this work, a process is presented to integrate aerogels in microchannels with reproducibility, mechanical stability, and no shrinkage. This protocol is based on the filling of channels during the gelation, which is crucial to avoid shrinkage, CO_2_ supercritical drying, and mechanical additives (polyethylene glycol and carbon nanotubes). Furthermore, the influence of polyethylene glycol and carbon nanotubes on the compressive strength, porosity, and specific surface area is investigated. Following the suggested process protocol, the integration of different aerogel compositions (with and without reinforcement) is successfully achieved in the microchannels without shrinkage and cracks. This research opens up new possibilities for the use of different aerogels in microfluidics with structural integrity and enhanced functionality.

## 1 Introduction

Silica aerogels were first fabricated by Samuel Kistler in the 1930s using a sol–gel technique followed by supercritical drying.^1^ These are highly porous materials with unique properties such as high specific surface area (up to 1200 m^2^ /g), high thermal insulation (0.005 W/m K), low density (down to 0.003 g/cm^3^), high porosity (up to 99.8%), and low dielectric constant (*k* = 1.0–2).^2−4^ Generally, aerogel fabrication consists of three major steps: starting of a sol–gel process, aging of the gel, and subsequently drying.^1−6^ The solution is prepared by adding the starting materials together with the catalysts to achieve the gelation. Commonly, the precursors are alkoxides with a high degree of purity such as tetramethyl orthosilicate (TMOS) and tetraethyl orthosilicate (TEOS).^2,5,6^ The aging step is performed to strengthen the gel network. This step involves several mechanisms such as condensation, dissolution, and reprecipitation of particles, resulting in a porous structure with the pores filled with the solution.^2−4,6,7^ The next step is drying, when the solution is removed from the pores and the final aerogel is obtained. The drying can be done using different techniques: ambient drying, supercritical drying, or freeze-drying.^4^ This is a critical step as unwanted shrinkage can occur due to capillary pressure.^2,8,9^

As a result of its unique properties, aerogels have several potential applications, for example: as catalyst support or carrier material in pharmaceutic industry, medical implants, cosmetics, and thermal insulation.^10−17^ Silica aerogels have also been studied as porous medium for several microfluidics applications from biochips to insulators.^18−30^ As an example, aerogels were explored in biochips as a three-dimensional support structure for recognition of nucleotide acids.^23^ Porous materials present the advantage of a 3D internal surface area that immobilizes probes, capturing biological targets more efficiently than planar. Yet, porous materials such as hydrogel present low thermal and mechanical stability. Therefore, aerogels can be advantageous in this regard. Other porous materials, such as porous ceramic micro struts, were also explored as a substrate for enzymatic reactions in microfluidic systems but present a lower specific surface area than aerogels (14.3 m^2^/g).^31^ Silica aerogels were also investigated as a thermal insulator in microelectromechanical systems (MEMS). In this example, the aerogels were used to improve the performance of micro hot plates by decreasing the utilized area to maintain the temperature and power consumed.^21^ One of the main challenges in the use of aerogel in microfluidic applications is its integration into the chips. One of the most common integration methods is the use of ground particles. For instance, aerogels were integrated as ground particles as a chromatography stationary phase into polydimethylsiloxane (PDMS) chips.^19^ Also, the use of aerogel thin films for obtaining micro bridges and cantilevers using lithography techniques was previously reported.^18^ Lastly, in a previous work, we reported the integration of silica aerogel monoliths in 1 mm-diameter holes, drilled in a glass wafer using ambient drying.^28^ For the gels to resist ambient drying without structure collapse, the mechanical stability was improved by using poly(ethylene glycol) (PEG) and hexamethyldisilazane (HMDS). Other types of aerogels were also explored in sensor applications.^20,22,30,32,33^ One example is graphene-based aerogels that were used for chemiresistive gas sensors. In that work, the sensitivity of the aerogel to oxidizing gases such as NO_2_ and W_18_O_49_ was improved by the addition of nanowires.^30^

Aerogels have an extensive range of applications in microtechnology and microfluidic devices. Yet, their integration is still minimal by the use of only ground particles and thin films. The integration of aerogels as a monolith in microfluidic channels has not been reported in the literature yet. This is in part due to the complexity of the process and the intrinsic shrinkage that leads to incomplete integration and adhesion between the aerogel and the chip. Another issue is the mechanical stability of aerogel, which is generally very fragile due to the pore structure and shrinkage cracks. This can be a limitation when aiming to fill microfluidic channels completely because the collapse of the aerogel structure can create voids into the channels that act as shortcuts when pumping liquids or gases. Moreover, their fragility can limit its reproducibility.

To allow the use of aerogels in microfluidics, it is crucial to obtain a process to incorporate aerogel monoliths in microchannels without shrinkage and high mechanical stability. In this study, the implementation of monolithic silica aerogels in closed microfluidic channels is presented in detail.

Our process covers the synthesis, integration, aging, and drying of the gels without shrinkage inside the channel. A CO_2_ supercritical drying technique was used to obtain aerogels without shrinkage and cracks, at low temperature and pressure (the critical point is at 35 °C and 85 bar).^2,9^ In this process, the temperature and pressure are above the critical point so the CO_2_ and ethanol are fully miscible and the solvents can be removed from the pores without damaging the structure.^2,8^ Additionally, different mechanical reinforcement strategies are investigated, namely, PEG, HMDS, and carbon nanotubes (CNTs), to improve the stability and compatibility of the aerogels for a broader range of applications. These strategies were already described in literature and present promising results in increasing the mechanical stability of monolith aerogels.^28,34,35^ The realized materials are then characterized in terms of chip integration (filling without shrinkage), porosity, and mechanical properties.

## 2 Materials and Methods

### 2.1 Chip Manufacturing

**Figure 1:**
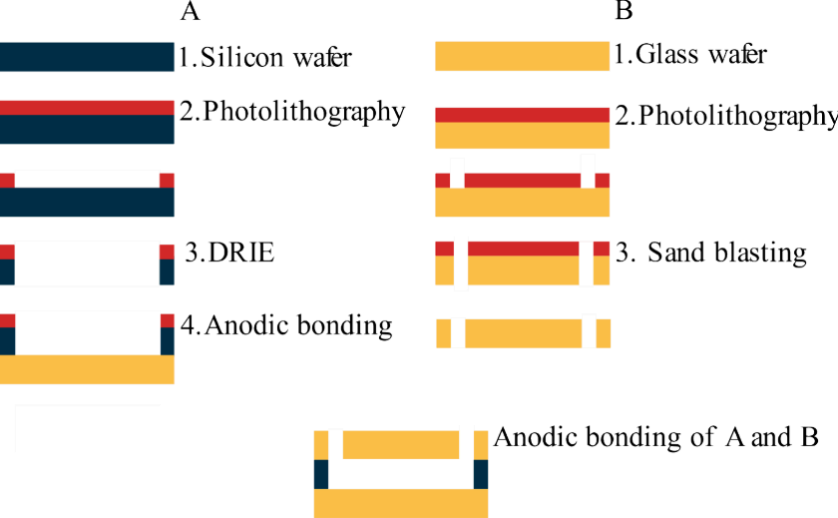
Process steps for chip manufacturing. Process route A shows the process steps of the silicon wafer, followed by anodic bonding with the bottom glass wafer. Process route B shows the process steps of the top glass wafer with inlets and outlets. Lastly, the wafers are bonded.

The used chips consist of a glass–silicon–glass chip with channel dimensions of 400 μm width, 380 μm height, and 7 mm length. The channel dimensions are based on the proportions commonly used in microfluidics. First, the photolithography process is performed in the silicon wafer to structure the channels. The resist (AZ10XT) is spin coated, and after exposure, the channels are etched using a deep reactive ion etching technique (DRIE). For the inlets and outlets, first a dry film photoresist, i-HE, is laminated in the glass wafer. After the development of the windows for etching the holes, with pressurized hot water for 3–5 min, they are sandblasted. The resists are removed, and the wafers are cleaned using piranha solution and then bonded via an anodic bonding technique. Finally, the chips are diced. The overview of the chip realization is shown in Figure 1.

### 2.2 Aerogel Synthesis and Chip Integration

The aerogels are synthesized via a sol–gel technique based on a two-step catalysis previously reported by us.^28^ In this study, the composition with the PEG additive remains from the past work, and the synthesis is adjusted for four new compositions: pure aerogel and aerogel with 2, 5, and 8 wt % CNTs. The additives (PEG and CNTs) are investigated based on their extensive use in literature as mechanical enhancers for aerogel's mechanical properties.^28,33−42^ Compared to the previous work, a different drying technique is also proposed: CO_2_ supercritical drying, from which aerogels without shrinkage and high reproducibility are obtained in closed microchannels. The samples' compositions are summarized in Table 1.

**Table 1:** Sample Names and Compositions^a^

	**composition**								
	**silica aerogel**								
	**molar ratio**								**wt %**
			**acid solution**		**basic solution**				
**name**	**TEOS**	**C_2_H_6_O**	**H**_**2**_**O**	**HCl**	**H**_**2**_**O**	**NaH**_**3**_	**HMDS**	**PEG**	**CNT**
**Si_aero**	1	5.5	1	7.8 × 10^–4^	2.5	5.7 × 10^–3^	0.36		
**Si_aero_PEG**	1	5.5	1	7.8 × 10^–4^	2.5	9 × 10^–3^	0.36	1.8 × 10^–4^	
**Si_aero_2CNT**	1	5.5	1	7.8 × 10^–4^	2.5	5.7 × 10^–3^	0.36		2 wt %
**Si_aero_5CNT**	1	5.5	1	7.8 × 10^–4^	2.5	5.7 × 10^–3^	0.36		5 wt %
**Si_aero_8CNT**	1	5.5	1	7.8 × 10^–4^	2.5	5.7 × 10^–3^	0.36		8 wt %

a All of the samples are made of silica aerogel with an HMDS coating, with or without additives.

First, a mixture of the alkoxysilane precursor TEOS (Sigma-Aldrich, for synthesis), ethanol (Th Geyer, 99.7%), and the acid solution (HCl/H_2_O) is stirred for 1.5 h at 60 °C in a water bath. The additives PEG (Sigma-Aldrich) or CNTs (multiwalled, 50–90 nm diameter, Sigma-Aldrich) are added into the mixture of TEOS and ethanol before the acid solution. During gel preparation, it was observed that the CNTs interfere with gelation, resulting in higher gelation times for those samples. To avoid the precipitation of the CNTs and creation of microcracks, it is important to speed up the gelation.^43^ To control the gelation, the basic molar ratio (NaH_3_/H_2_O) of those samples was adjusted for the ratios shown in Table 1. In addition to that, the CNTs were first added to the solution (TEOS/ethanol) and then placed in an ultrasonic bath for 15 min. Furthermore, the process was done under magnetic stirring until gelation was achieved to improve the CNT dispersion. As PEG is soluble in water, an ultrasonic bath was not needed.

Furthermore, the basic solution NH_3_/H_2_O is added, and the temperature is set to 40 °C. The gelation is obtained after 40 min for pure silica and PEG samples. For 2 and 5% CNTs, the gelation starts after 50 min, and for 8% CNTS after approximately 80 min. To produce monolithic samples for pore and mechanical characterization, 5 mL syringes are used as molds. The molds are filled shortly before the gelation and stored inside the oven (40 °C).

The complete aging process has a duration of 1 week. During the experiments, it was observed that speeding the aging process leads to pore collapse (powder aerogel). This can result from a short period for the aging mechanisms to be completed, e.g., redispersion of Si nanoparticles that reinforce the network. First, the gel is washed in a solution with a proportion of 70% volume ethanol/30% volume water at 50 °C for 1 day. The aerogel is aged first in 70% volume TEOS/30% volume ethanol at 70 °C for 3 days and then in pure ethanol at 50 °C for 1 day.

After aging, the hydrophobic coating is done for all the samples, and the gels are exposed to a solution of cyclohexane and HMDS for 1 day at 50 °C to obtain a hydrophobic coating. In a previous work,^28^ the contact angle of the Si_aero_PEG sample was determined to be 136°. Here, the new compositions were characterized and similar contact angles were found for the aerogel blocks. Lastly, the gels are washed in ethanol to remove any subproducts from this step, e.g., ammonia. Before drying, the pH is checked to make sure no ammonia is left.

**Scheme 1:**
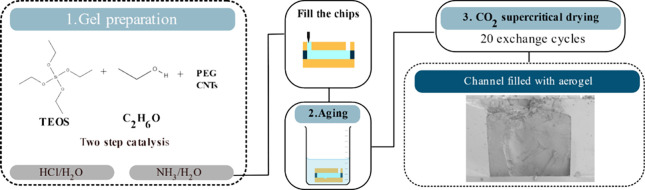
Process Overview First, the synthesis is done by a two-step catalysis using TEOS as the alkoxysilane precursor. The microchannel, with dimensions of 400 μm width, 380 μm height, and 7 mm length, is then filled and aged for a week. Finally, the chips are dried in a supercritical drying process.

The final step is drying of the gels to obtain the aerogels. The drying is performed in low-temperature supercritical drying equipment (Leica CPD300). We propose a 20-exchange cycle as the optimal number of cycles for removing ethanol completely from the pores. It was observed that for such a sample with high porosity, a smaller number of cycles can result in ethanol leftovers that create pore collapse (inside microfluidic channels and monolithic aerogels). The ethanol and CO_2_ are mixed for 30 min before the exchange cycles start, and 20 exchange cycles are done. The complete process takes around 2 h. The complete process is shown in Scheme 1.

### 2.3 Pore Characterization

Silica aerogels are well known as highly porous materials (up to 99%) and typically present mesoporosity with porous sizes from 1 to 100 nm.^1^ Aerogels also present an unusual combination of high porosity and small pore size, which makes the usual porosity characterization methods difficult to be performed, e.g., mercury intrusion. Despite the difficulties of characterizing such materials, the most widely used methods are nitrogen adsorption/desorption and BET to obtain the isotherm shapes and specific surface area. However, high-resolution SEM and TEM can also be limited.^2^ Here, the samples’ porous characteristics are investigated via nitrogen adsorption/desorption, BET, BJH, and SEM.

The samples' specific surface area and average pore diameter are obtained by a nitrogen adsorption technique at 77 K. Before the analysis, the samples are ground and heated at 70 °C for at least 1 day, to evaporate any adsorbed vapors. The specific surface area and the average pore size are calculated using the methods Brunauer–Emmett–Teller (BET) eq 1, and Barrett–Joyner–Halenda (BJH) eq 2, respectively.^44,45^
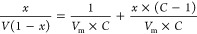
1*V* is the volume absorbed by molecules, *V*_m_ is the monolayer volume, *C* is a constant, and *x* is the relative pressure (*P*/*P*_0_).

The BJH method estimated the pore size distribution. The calculation assumes that the pore has a cylindrical shape and that the absorbed amount is related to physical absorption and capillary condensation in the pores using the classical Kelvin eq 2. The BJH method calculates the change in the thickness of the absorbed film from the decrease of relative pressure in the desorption.^44,46^
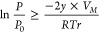
2where *r* is the pore radio, *P*/*P*_0_ is the relative pressure in equilibrium, *y* is the surface tension of the adsorbate in liquid form, *V*_M_ is the molar volume of the liquid inside the pores, *T* is temperature, and *R* is the universal gas constant.

In addition to the nitrogen adsorption measurements, micrographs of the aerogels were obtained by scanning electron microscopy (Zeiss) using secondary electron (SE2) and In-lens modes. With this technique, the aerogel porous structure was investigated as well as the dispersion of the CNTs in the aerogel matrix. The cross sections of the microchannels were also analyzed to confirm that there was no shrinkage inside the channel and no openings are created. For the preparation, the chips are cut by scratching the glass with a diamond pen and then broken by a mechanical bending force.

### 2.4 Mechanical Tests

The mechanical properties of the produced monolithic aerogels were characterized by compression tests following the standard ASTM C133. For that, cylindrical samples with a diameter of 12 mm were prepared using 5 mL syringes as molds. Both ends of the samples were ground with 1200 mesh sandpaper to obtain flat and parallel surfaces. The height-to-diameter ratio was about 1.5:1 for most samples with the exception of samples with 8 wt % CNTs. These samples showed precipitation of part of the CNTs and areas of pure silica, which were removed during sample preparation, resulting in a ratio of about 1:1. The tests were conducted using a universal testing machine, Zwick Z005 (Zwick Roell Group, Ulm, Germany). To account for possible nonparallelism of the samples, a half sphere was used to apply the load. A total of five samples per condition were tested until failure with a loading speed of 1 mm/min. The fracture of the samples during the mechanical loading was recorded by using a FOculus F0531C digital camera (New Electronic Technology Vertriebsgesellschaft mbH, Finning, Germany) synchronized with the testing machine.

## 3 Results and Discussion

All the samples were characterized in terms of chip integration, porosity, and mechanical properties. In this section, the sample names, presented in Table 1, will be used to refer to the sample’s compositions. In the following section, the aerogel synthesis steps (gel preparation, aging, and drying) are discussed in detail, focusing on aerogels without shrinkage and high reproducibility.

The influence of the reinforced materials on the silica aerogel microstructure is also investigated in the SEM, N_2_ physisorption, and mechanical test analyses. The synthesis of hybrid silica compounds using organic polymers with polar groups such as PEG and embedded solid phases such as CNTs is well documented in the literature. Consequently, the chemical interactions between such precursors have already been studied in detail. Overall literature shows that such chemical and physical bonds are difficult or impossible to detect with IR instruments, which is why we did not aim to detect these bonds in detail in the present work.^1,47−51^ Rather, this work focuses on the resulting material properties, which are consistent with the presence of such bonds.

### 3.1 Chip Integration

**Figure 2:**
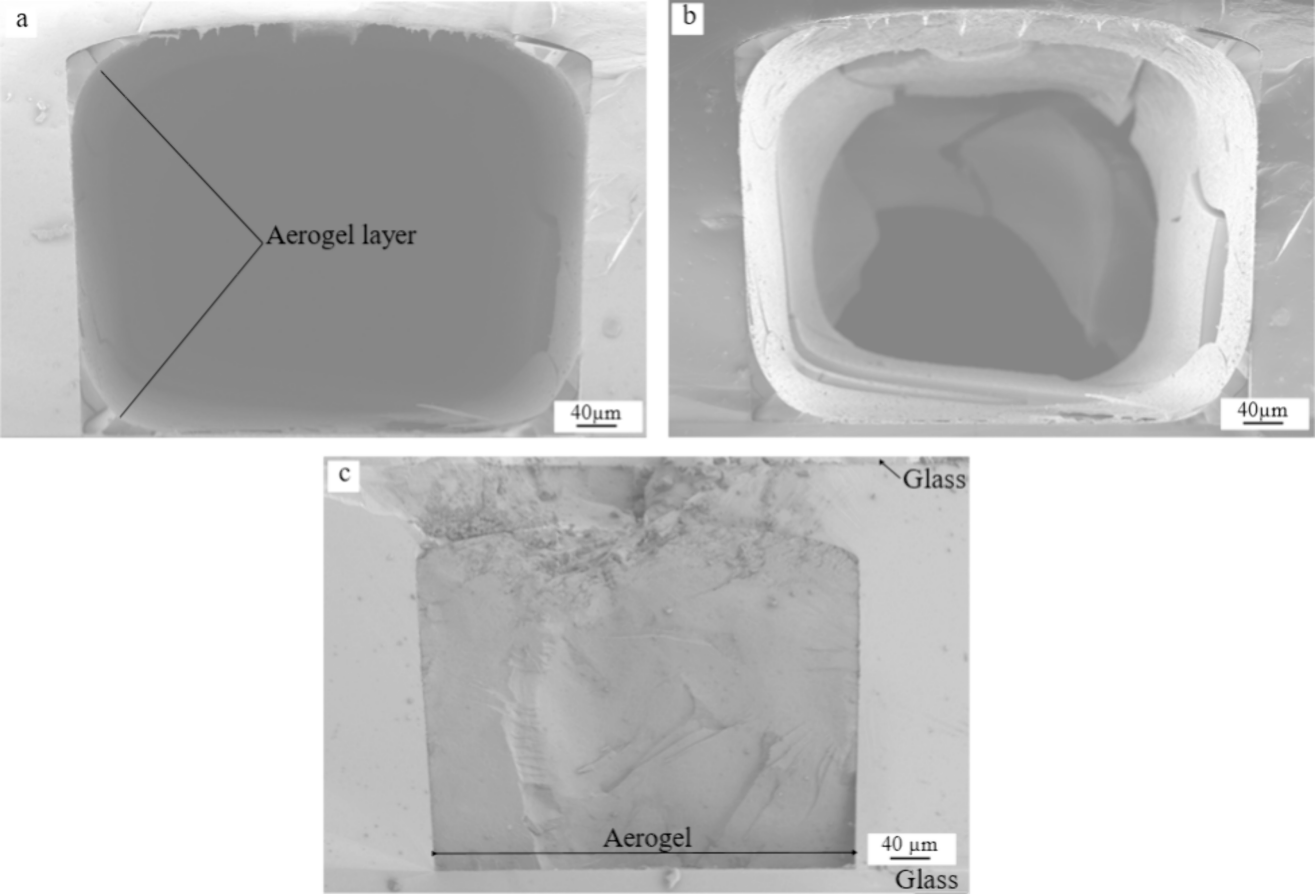
(a) Cross section of the chip filled with Si_aero before gelation; only a thin aerogel layer remains. (b) The same chip is investigated with mixing of signals (In-lens and SE2), and it is observed that the thin layer presents low quality with cracks along the channel. (c) Cross section of the chip filled with Si_aero during gelation; the aerogel remains intact even without reinforcement.

**Figure 3:**
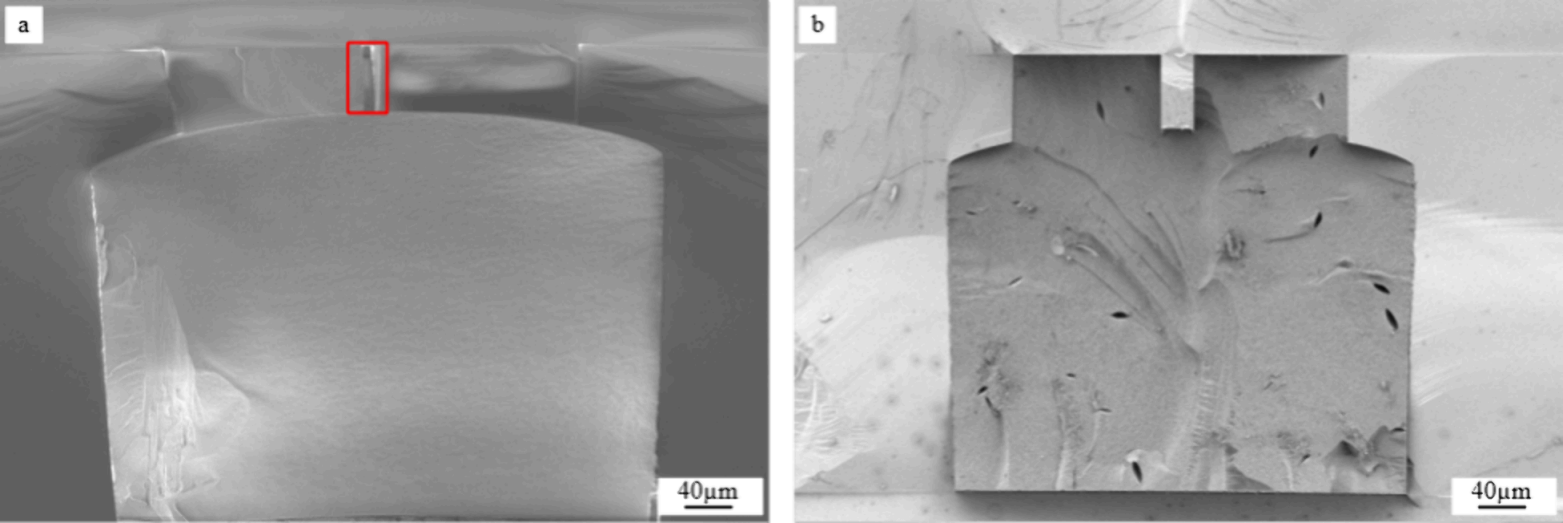
(a) Cross section of the channel with a highlighted 50 μm high microwall, filled with Si_aero. (b) Cross section of the chip filled with Si_aero_8CNT. Both samples show that different channel geometries can be completely filled with aerogel following the proposed protocol, without cracks and shrinkage.

The cross sections of the aerogel-filled microchannels were analyzed by SEM and are shown in Figures 2 and 3. As previously mentioned, the silicon chips were scratched and broken with bending force. This preparation method was preferred to preserve the aerogel’s structure and to obtain a clean, cleaved surface. The other common preparation method used in MEMS technology for such purpose would be sawing, but that results in pronounced sawing marks in the cross section, and the cooling water used in the process could damage the aerogel’s porous structure. The results of the chosen preparation method are shown in both Figures 2 and 3. Although some marks resulting of the mechanical stress can be still seen in the aerogel and in the chip, the overall cross sections are smooth in the SEM images.

By SEM analysis, it is possible to observe the influence of the filling time of the channel in the final result of the aerogels. In Figure 2a, the sample was filled with the silicon aerogel solution before gelation. It is seen that the aerogel collapsed, and only an aerogel layer remained on the chip walls. During gelation, a change in the viscosity happens and the gel shrinks; therefore, it is possible that an aerogel powder was also obtained but lost during the sample preparation (chip break), and only the layer attached to the walls was stable enough.

A deeper analysis is done in the same sample by combining two signals in the SEM (In-lens and SE2), displaying both the front of the chip and the inside of the channel in Figure 2b. In this analysis, it is observed that the aerogel thin layer covers all the walls of the chip, including the end of the channel. It is also observed that the aerogel layer presents low quality, as several cracks are present, and part of the layer is detached at the end of the chip.

In contrast, when the chip was filled with Si_aero during gelation, the sample showed no shrinkage or cracks even without reinforcement, as shown in Figure 2c. Some damage in the silicon chip is observed on the top of the aerogel, which is caused by the mechanical distress during sample preparation. Clearly, the aerogel remains intact.

A different channel design was tested to evaluate whether the protocol also works with more complex geometries. A channel containing a 30 μm-thick and 50 μm-high microwall was filled with the aerogel applying the protocol discussed. Figure 3a shows that the channel is completely filled with Si_aero, also in the areas around the microwall. No shrinkage or cracks were observed. Here, the microwall needed to be highlighted in red as the samples presented lower contrast if compared with Figure 3b.

For comparison, Figure 3b presents the cross section of a chip with Si_aero_8CNT, i.e., the sample with the highest CNT concentration. Also, in this case, the channel is filled without defects. The presence of CNTs did not seem to interfere with the integration of the aerogels inside any of the microfluidic channel geometries investigated.

In sum, the final quality of silica aerogels in closed microfluidic channels is highly dependent on the filling time of the gels. This step needs to be performed during gelation to avoid shrinking, as shown in Figure 2. As mentioned in Section 2.2, a supercritical drying technique such as CO_2_ supercritical drying enables the control of the shrinkage during this critical step. To be performed correctly, the number of exchange cycles between ethanol and CO_2_ was investigated and set to 20 to avoid leftovers in the porous structure, which could further lead to pore collapse. Following the protocol established in this research, all the samples presented the same positive result with an aerogel-filled channel without shrinkage, leaks, or cracks, regardless of the microchannel geometry. We also observed in the micrographs and visual inspection during the handling of the chips that all the aerogels seem to present similar adhesion to the chip walls.

### 3.2 Pore Characterization

**Figure 4:**
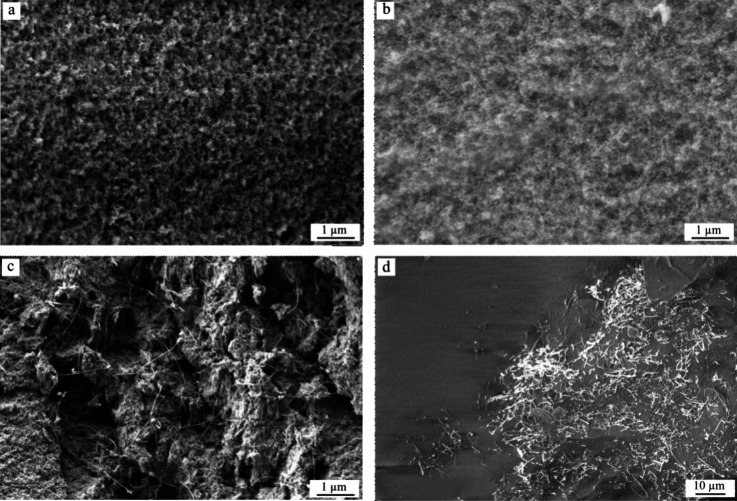
(a) Silica aerogel porous structure. (b) Silica aerogel reinforced with a PEG porous structure. (c) Silica aerogel with 8% CNTs, and CNTs are deposited through the pores. (d) Si_aero_5CNT top view with small magnitude; CNTs are not spread uniformly in the matrix.

The pore structure of the samples was analyzed by both SEM and a nitrogen adsorption technique. A SEM micrograph of sample Si_aero is shown in Figure 4a, and the Si_aero_PEG micrograph is shown in Figure 4b. The obtained micrographs show the aerogel's highly porous network, as expected by the presented literature. The pore size is further analyzed with nitrogen adsorption, but it can be seen from both micrographs that the pores present a rounded shape and no preferential growing direction. Additionally, no crack was observed in the structures in the SEM. The micrographs of both Si_aero and Si_aero_PEG present similar results regarding the SEM analysis.

In Figure 4c,d, the micrographs show the CNT's dispersion in the aerogel. In Figure 4c, it is observed that the CNT is dispersed untidily through the pore network, not lying on the surface or being part of the network itself.

With a smaller magnification, it is possible to observe that the CNTs are also not deposited uniformly throughout the network, as shown in Figure 4d. As previously mentioned, we tried to improve the distribution by external forces, such as ultrasonic bath and control of the gelation time. Moreover, the molds and chips were filled shortly before gelation to avoid the CNT precipitation. The precipitation of the CNTs was also observed previously in literature and, if too pronounced, can lead to microcracks that influence negatively the mechanical properties of the aerogel.^43^ A nonuniform dispersion of the CNT is observed also in our samples, but no microcracks were observed in the micrographs, also in the regions with higher concentration of CNTs. This is probably because of the proposed protocol that makes use of several strategies, i.e., ultrasound, magnetic stirring, and control of gelation time. It controls the precipitation of the CNT and further creation of the microcracks.

**Figure 5:**
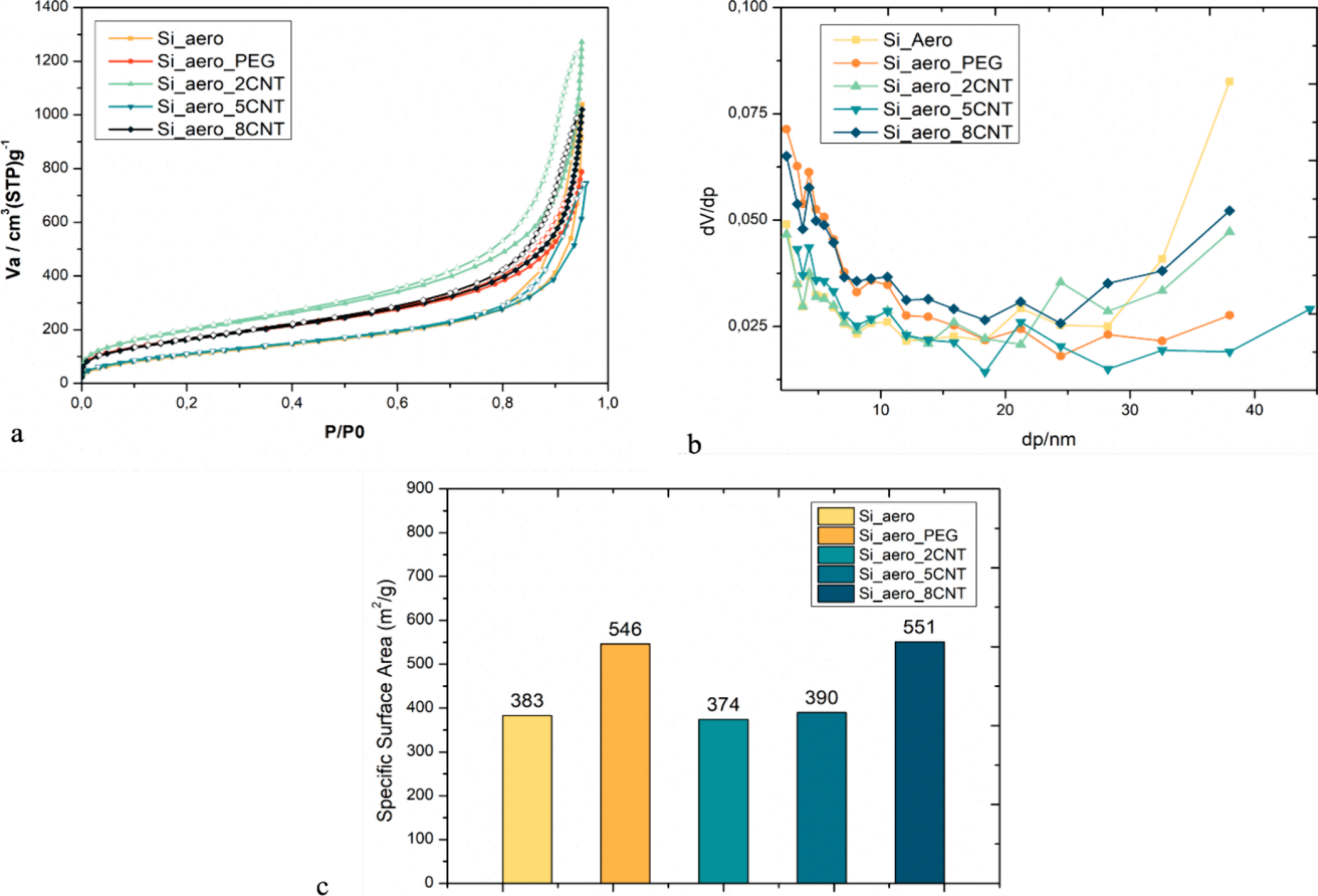
(a) Adsorption/desorption isotherm curves (adsorption data: filled symbols, desorption data: open symbols). (b) Pore volume distributions and (c) specific surface areas of the samples analyzed by nitrogen physisorption.

The investigation of the pore structure was carried out using a nitrogen adsorption technique with ground samples. First, the adsorption–desorption isotherms were determined (seen in Figure 5 a). All the samples examined had the typical shape of mesoporous materials, which is also confirmed by BET and BJH analyses of the isotherms. Furthermore, the pore volume distributions are shown in Figure 5b, and the specific surface area is shown in Figure 5c. Correlating both pictures, it can be observed that the SSA results follow the pore volume distributions of micro and meso pores (*d* < 10 nm), as the larger pores do not contribute significantly to the specific surface area results.

From the nitrogen adsorption analysis, the highest specific surface area (SSA) of 551 m^2^/g was obtained for the silica aerogel with 8% CNT (Figure 5c). The same sample presents the highest volume of small pores. For the PEG-reinforced sample, the relative volume of micropores (*d* < 2 nm) is similar to the one observed for 8% CNT, while a slight decrease in the relative volume of the mesopores (*d* = 10–20 nm) is observed. For this reason, the SSA values obtained for these two samples do not vary significantly. For the PEG sample, the increase of the SSA due to the presence of smaller pores compared to pure aerogel was already expected. This behavior is also seen in literature, where polymers such as PEG are used to tailor the pore size of aerogels by means of controlling the rate of polymerization of the silica oligomers and the type of polymeric interactions within the network.^52,53^ For example, a study was performed with silica aerogel reinforced with 10–40 wt % of PEG.^52^ There, the average pore size and SSA could be tailored by means of PEG content; while the SSA increased with a PEG amount between 10 and 20 wt %, it decreased significantly with higher contents of PEG (between 20 and 40 wt %). Because the PEG content used in the present work is relatively small, an increase in the SSA was expected.

The samples pure aerogel and aerogel with the lower CNT contents, 2 and 5 wt %, showed similar values in the SSA. Here, it is also observed that the relative volume of smaller pores is less pronounced compared to the other samples. Comparing the SSA and relative pore volume values of the CNT-reinforced sample, it is possible to observe that there it is not drastically changed, but a higher presence of smaller pores and an increase of SSA are observed with higher contents of CNTs. Similar studies with CNT contents also observed the same behavior. For instance, an investigation performed with silica aerogel samples doped with CNT contents between 0.1 and 2.5 wt %^47^ observed a small variation in the SSA and in the density for all the samples with the CNT. The study presented a decrease of the SSA for the samples with the lowest concentration (0.1 wt %) compared to pure aerogel, followed by a small increase of the SSA and density with the increase of CNT contents. The authors hypothesized that the CNTs could influence the pH of the solution during synthesis, which could lead to a change in the SSA and density of the final material.

Overall, the different aerogel compositions of this work presented higher SSA compared to other porous materials used in microchannels such as porous ceramic micro struts (14.3 m^2^/g)^31^ and silicon nanowires 342 m^2^/g.^54^ Other porous materials, such as hydrogels, commonly used in BioMEMS (biological micro electromechanical systems), can also achieve higher surface areas, up to 423 m^2^/g,^55^ but present lower mechanical stability compared to aerogels.

### 3.3 Compressive Test

**Figure 6:**
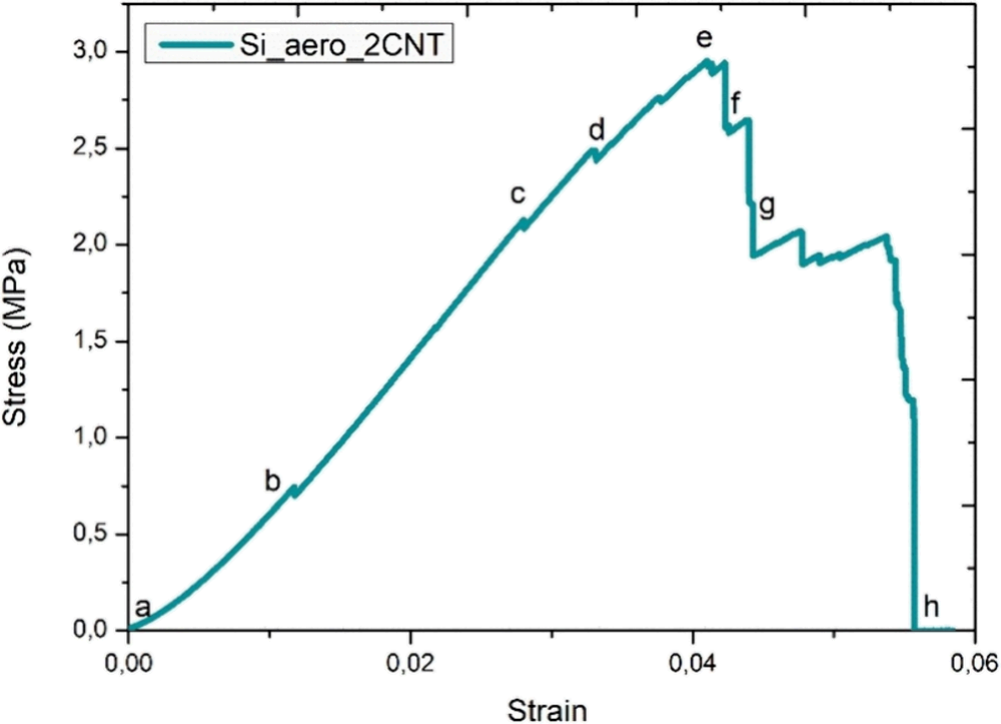
Example stress–strain of an aerogel sample with 2 wt % CNT.

**Figure 7:**
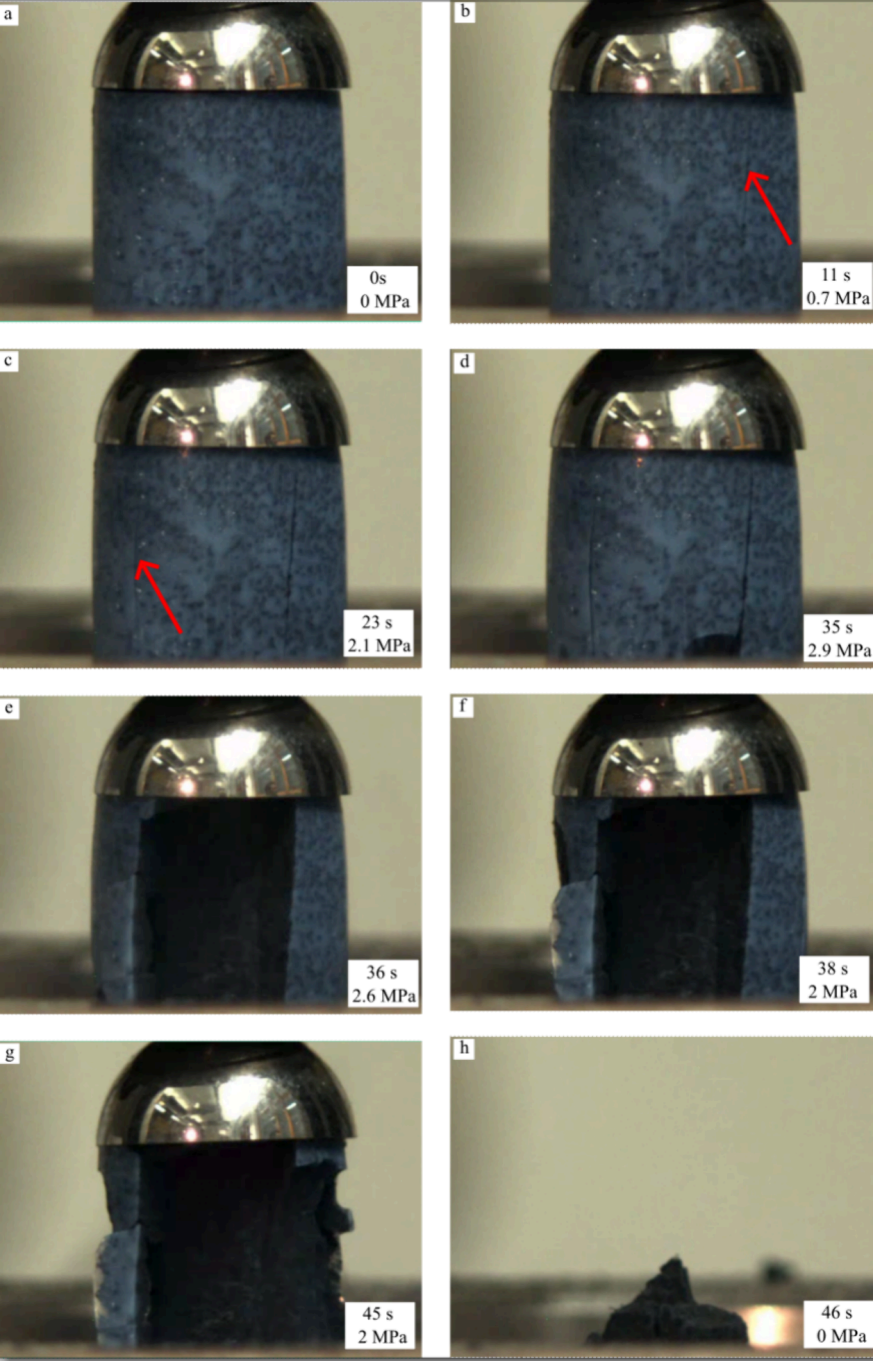
View of the Si_aero_2CNT sample during the compressive test at different loading stages. The pictures (a–h) were taken during the compressive test and correspond to the force drops presented in Figure 6a–h.

The mechanical performance of the produced aerogels was evaluated by compressive tests. The stress–strain response of sample Si_aero_2CNT is shown in Figure 6 as an example. The sample shows mostly linear elastic deformation with several small load drops starting at 0.7 MPa. Aerogels are highly porous materials; therefore, the mechanical load is sustained throughout the thin solid matrix of the material during a compressive test. The observed load drops represent the failure of different struts in the solid matrix when the local stress is too high due to the nonuniform structure. However, these initial load drops do not lead to the catastrophic final failure of the material, which happens at a much higher deformation. Instead, as each strut fails, the generated cracks are stopped in the porous network. As a result, part of the aerogel collapses, dissipating mechanical energy, while the remaining structure can still sustain the load. For reference, Figure 7 shows pictures of the same sample taken at different times during the compressive test and can be correlated with the force drops in Figure 6. The red arrows in Figure 7b,c indicate the starting cracks in the sample. A similar behavior is observed for all samples, independent of the type of reinforcement.

**Figure 8:**
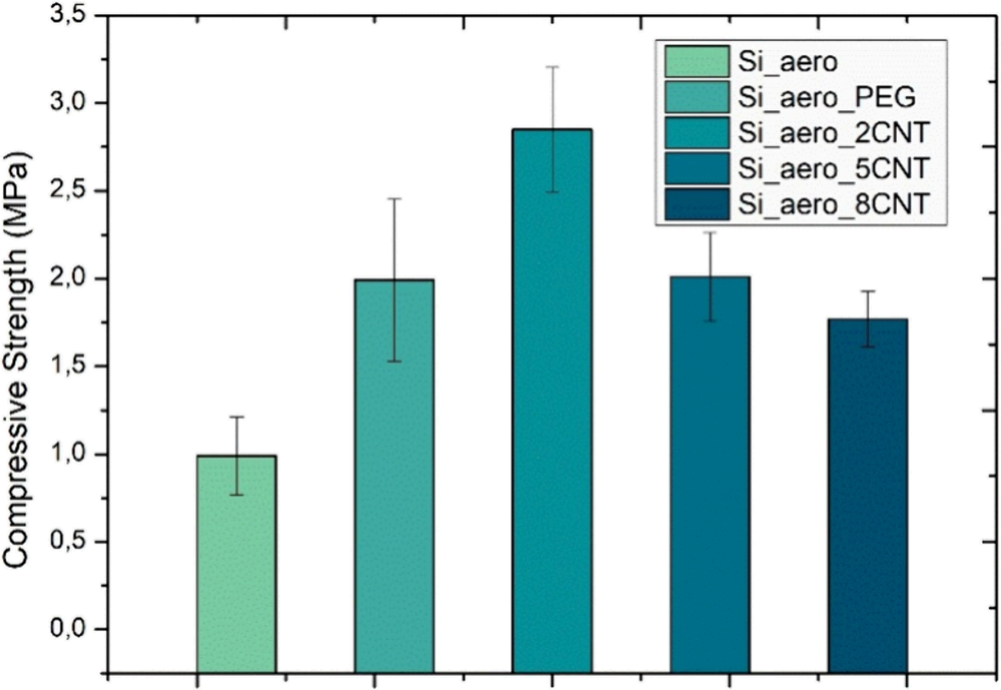
Compressive strength of silica aerogel samples with and without reinforcement. Averages and standard deviations were calculated from at least five samples per condition.

The results of the compressive tests in terms of compressive strength are summarized in Figure 8. As expected, the pure aerogel samples show the lowest compressive strength of 1.0 ± 0.3 MPa. With the addition of PEG, the compressive strength increases to 2.0 ± 0.5 MPa. It has been reported that even small amounts of PEG can lead to increase of stiffness and mechanical stability of the aerogel’s solid matrix,^34^ hence resulting in the observed strength increase. The introduction of CNTs also leads to an increase in strength, although the underlying mechanism is different. Instead of directly altering the stability of the solid matrix, the CNTs act as a second-phase reinforcement dispersed along the structure of the aerogel. Due to the higher stiffness and strength of CNTs, an overall increase of strength is to be expected. In this regard, the strengthening will depend on the amount, dispersion, and orientation in relation to the load of the CNTs. There seems to be an optimum amount for CNTs of 2 wt % while higher amounts lead to lower strength values. This behavior was already reported in literature, where the best mechanical behavior was obtained for samples with lower CNT loadings (0.5–1 w.t%), with a maximum compressive strength of 0.9 MPa.^47^ Here, a maximum compressive strength value of 2.9 ± 0.4 MPa is obtained for samples of 2%. It is hypothesized that the higher heterogeneity of the samples with higher amounts of CNTs can result in regions that are more fragile. In other words, since the stiffness of the structure is not constant, higher deformation is expected to happen in the regions with low concentration of CNTs, causing their earlier failure. As these regions fail at lower stresses, the remaining structure experiences higher stress concentration, leading to a lower measured strength in comparison to the samples with lower concentrations of CNTs.

It is important to observe that all the reinforced samples increased the aerogel′s mechanical properties and were successfully integrated on the chip and therefore can be wildly explored in applications that require more mechanical stability, e.g., flow sensors.

## 4 Conclusions

For the first time, aerogels were successfully integrated in closed microfluidic channels by developing a process that prevents shrinkage, resulting in wholly filled channels. The process steps were investigated, resulting in a protocol for the realization of different aerogels with and without mechanical reinforcement. Some major points observed were the filling of the channels with the gels during gelation, 1-week aging of the gel, and a supercritical drying process with 20 exchange cycles between ethanol and CO_2_. We investigated and proved that the integration of the gel during gelation prevents the shrinkage in the chip, resulting in a completely filled channel. The aging step and the number of exchange cycles are equally important, as during aging, the silica network is strengthening; moreover, an insufficient number of exchange cycle between ethanol and CO_2_ can lead to ethanol leftovers inside the pores that further result in a pore collapse. The aerogels were also successfully reinforced by PEG and CNTs (2, 5, and 8 wt %), with an increase of the compressive strength up to three times higher than for pure silica aerogels. The use of such additives also resulted in a tailorable high SSA, being the two highest values for PEG and 8% CNT. The PEG alters the stability of the solid matrix itself and tailors the pore size, while the CNTs act as a secondary reinforced phase in the system. Nevertheless, the amount of the added reinforcement is very important; too high amounts of CNTs can lead to heterogeneity of the material and decrease of strength, as previously seen in literature. Overall, all of the recipes were successfully integrated in microfluidic channels with different geometries, enabling the use of different aerogels in microfluidics with improved structural integrity and a great potential for an enhanced functionality. This opens a different range of possibilities for the use of aerogels in microfluidics such as thermal insulation, BioMEMS, and catalysis.
